# The Clinical Utility of SUDOSCAN in Chronic Kidney Disease in Chinese Patients with Type 2 Diabetes

**DOI:** 10.1371/journal.pone.0134981

**Published:** 2015-08-13

**Authors:** Andrea O. Y. Luk, Wai-Chi Fu, Xue Li, Risa Ozaki, Harriet H. Y. Chung, Rebecca Y. M. Wong, Wing-Yee So, Francis C. C. Chow, Juliana C. N. Chan

**Affiliations:** 1 Asia Diabetes Foundation, Prince of Wales Hospital, Hong Kong, SAR; 2 Hong Kong Institute of Diabetes and Obesity, Prince of Wales Hospital, Hong Kong, SAR; 3 Department of Medicine and Therapeutics, The Chinese University of Hong Kong, Hong Kong, SAR; 4 International Diabetes Centre of Education, The Chinese University of Hong Kong, Prince of Wales Hospital, Shatin, Hong Kong, SAR; Madras Diabetes Research Foundation, INDIA

## Abstract

There are gaps between recommendations on regular screening for diabetic kidney disease (DKD) and clinical practice especially in busy and low resource settings. SUDOSCAN (Impeto Medical, Paris, France) is a non-invasive technology for assessing sudomotor function using reverse iontophoresis and chronoamperometry which detects abnormal sweat gland function. Vasculopathy and neuropathy share common risk factors and we hypothesized that SUDOSCAN may be used to detect chronic kidney disease (CKD). Between 2012 and 2013, SUDOSCAN was performed in a consecutive cohort of 2833 Hong Kong Chinese adults with type 2 diabetes. Chronic kidney disease was defined as estimated glomerular filtration rate <60 ml/min/1.73m^2^. In this cross-sectional cohort (mean age 58.6±9.5 years, 55.7% male, median disease duration 8 [interquartile range 3–14] years), 5.8% had CKD. At a cut-off SUDOSCAN-DKD score of 53, the test had sensitivity of 76.7%, specificity of 63.4% and positive likelihood ratio of 2.1 to detect CKD. The area under receiver operating characteristic curve for CKD was 0.75 (95% confidence interval 0.72–0.79). Patients without CKD but low score had worse risk factors and complications than those with high score. We conclude that SUDOSCAN may be used to detect patients at risk of impaired renal function as part of a screening program in Chinese population, especially in outreach or low resource settings.

## Introduction

Diabetic kidney disease (DKD), indicated by albuminuria and/or reduced glomerular filtration rate (GFR) predict end-stage renal disease, cardiovascular disease and all-cause mortality [1,2]. Due to its insidious nature and lack of overt symptoms until the advanced stages, regular laboratory investigations are needed to detect DKD and its progression [3]. Major international guidelines recommend screening for DKD on an annual basis by measuring serum creatinine and urine albumin excretion [4]. With aggressive metabolic control and appropriate use of disease-modifying medications namely renin-angiotensin system (RAS) blockers, DKD is highly treatable [5,6]. Yet, many patients are not screened due to poor disease awareness, costs of tests and/or lack of infrastructure and personnel to support screening especially in low resource or busy clinic settings [7]. Consequently, a reliable, convenient and non-invasive technology which can identify subjects at risk of DKD may add value to these detection and preventive strategies.

SUDOSCAN (Impeto Medical, Paris, France) is a novel technology that provides precise evaluation of sudomotor function through reverse iontophoresis and chronoamperometry. The technology enables measurement of electrochemical skin conductance based on reaction between sweat chlorides and stainless-steel electrodes when low direct-current (DC) voltage is applied [8]. A further advantage of the SUDOSCAN is that measurements are unaffected by ambient heat or humidity [8]. Sweat glands are innervated by small unmyelinated sympathetic C nerve fibers [9] and sudomotor dysfunction is known to occur during early stage of diabetes and pre-diabetes [10]. The same device with different built-in algorithms (EZSCAN) has been used to predict diabetes by evaluating early deficits in sudomotor function, and previous studies have reported association of electrochemical skin conductance with glycated hemoglobin (HbA_1c_), fasting plasma glucose and 2-hour plasma glucose during oral glucose tolerance test [11–15].

Since autonomic neuropathy reflects microvascular damage due to hyperglycemia and related metabolic perturbation [16], the severity of sudomotor dysfunction may serve as a surrogate for other microvascular complications including nephropathy. In a proof of concept case-control study involving 50 Chinese patients with type 2 diabetes with renal complication (estimated GFR <60 ml/min/1.73m^2^ and albuminuria) and 50, without, SUDOSCAN-DKD score was highly correlated with GFR and albuminuria [17]. In the current study, we expanded the sample size to evaluate the clinical utility of SUDOSCAN in detecting impaired renal function and determine the score cut-point for detecting patients at risk of chronic kidney disease (CKD).

## Materials and Methods

### Patients and Settings

We enrolled patients from two diabetes centers in Hong Kong: the Diabetes and Endocrine Centre at the Prince of Wales Hospital, and, the Yao Chung Kit Diabetes Assessment Centre affiliated with the Chinese University of Hong Kong. Between 1^st^ June 2012 and 31^st^ October 2013, all Chinese adults aged ≥18 years with type 2 diabetes who presented to either center for assessment of diabetes complications, were invited to undergo additional evaluation using the SUDOSCAN. Patients were excluded if they had type 1 diabetes (presentation with ketoacidosis or requiring insulin within a year of diagnosis), or had amputation of an arm or a leg. Referral sources for the centers included private and public hospital-based and community clinics, as well as by self. All patients gave written informed consent and the study was approved by the Joint Chinese University of Hong Kong—New Territories East Cluster Clinical Research Ethics Committee. Declaration of Helsinki was adhered to.

### SUDOSCAN

SUDOSCAN is a patented device designed to assess sudomotor function based on reverse iontophoresis. This technology uses low DC stimulation to extract chloride ions from sweat to create a current when these electrically charged ions encounter specific electrodes. The current produced is proportional to the chloride concentration that reacts with the electrodes, which in turn reflects sweat gland function and its sympathetic innervation.

Four electrodes were placed on areas most enriched in sweat glands, namely the palmar sides of hands and plantar sides of feet. A DC current at an incremental voltage of less than 4 volts was applied and the conductance (measured in μS), expressed as a ratio of the current generated to the constant DC stimulus, was calculated for hands (left and right) and feet (left and right). A time/ampere curve was recorded for each deviation and the mean value was estimated for each area with low conductance correlating with high risk of sudomotor function abnormality.

During the test, the patient placed his or her hands and feet on the electrodes. All electrodes were connected to a computer for recording and data management. The test took 2 minutes to perform, was painless, and no subject preparation was required. In addition, SUDOSCAN had built-in algorithms which integrate electrochemical skin conductance with age to produce a score that estimates current risks of CKD (SUDOSCAN-DKD score) [17].

### Metabolic and Complication Assessment

All patients underwent clinical assessment as part of a quality improvement program using the web-based Joint Asia Diabetes Evaluation (JADE) portal [18,19]. The latter contained built in templates to guide comprehensive assessment of risk factors and diabetes complications followed by automatic risk stratification, personalized reporting and decision support. Clinical data included demographic, socio-economic and lifestyle factors as well as medical and family history, BPs and anthropometric parameters including height, weight and waist circumference were obtained using standard procedures. Fundi were assessed using retinal photography through dilated pupils. Retinopathy was confirmed by the presence of dot and blot haemorrhages, hard exudates, cotton wool spots, neovascularization, laser scars, or a history of vitrectomy. Peripheral sensory neuropathy was diagnosed by two of the three features: 1) reduced sensation to monofilament in any part of the sole with normal skin; 2) a score of ≤6/8 (age younger than 65 years old) or ≤4/8 (age older than 65 years old) using a 128-hertz graduated tuning fork and 3) self-report of abnormal sensation in lower limbs. All assessments were carried out by trained nurses while the interpretation of retinal photos was undertaken by diabetologists. Cardiovascular disease was defined as history of coronary heart disease, stroke or peripheral vascular disease, the latter defined as non-traumatic lower extremity amputation and/or ankle:brachial ratio less than 0.9 by Doppler ultrasound scan.

Blood and urine samples were collected for plasma glucose, HbA_1c_, total cholesterol, low-density lipoprotein (LDL)-cholesterol, high-density lipoprotein (HDL)-cholesterol, triglyceride, renal function test and urine albumin-to-creatinine ratio (ACR), after at least 8 hours of fasting. Estimated GFR as expressed in ml/min/1.73m^2^ was calculated using the abbreviated Modification of Diet in Renal Disease (MDRD) equation recalibrated for Chinese: estimated GFR = 186×[SCR × 0.011] ^-1.154^ × [age] ^-0.203^ × [0.742 if female] ×1.233, where SCR was serum creatinine in μmol/l and 1.233 was the adjusting coefficient for Chinese [20]. Chronic kidney disease was defined as estimated GFR<60 ml/min/1.73m^2^. Microalbuminuria was defined as urine ACR ≥2.5–25.0 mg/mmol in men and ≥3.5–25 in women, and macroalbuminuria, urine ACR ≥25.0 mg/mmol.

### Statistical Analysis

The objectives of our initiative to evaluate the efficacy of SUDOSCAN in detecting and predicting CKD were set to firstly examine the association of SUDOSCAN-DKD score with estimated GFR in a cross-sectional cohort, and at the next stage, to determine whether baseline SUDOSCAN-DKD score is associated with incident CKD in patients with normal renal function at baseline. We estimated the sample size based on expected incidence of CKD to enable future prospective analysis. Assuming a correlation r value of 0.4 between estimated GFR and SUDOSCAN-DKD score, 63 cases with CKD will give more than 95% power to confirm this correlation. Assuming an annual incidence of CKD of 6.0%, 1900 patients would confirm this incidence with 95% confidence interval of 5.0–7.2%. Assuming 30% of patients will not be returning for repeat GFR at 1 year, we recruited over 2800 patients in the present cohort.

All data were expressed as mean±standard deviation (SD), median (inter-quartile range) or percentages as appropriate. We used multiple linear regression to estimate the independent correlation of SUDOSCAN-DKD score with estimated GFR adjusted for gender, disease duration, body mass index (BMI), systolic blood pressure (BP), HbA_1c_, LDL-cholesterol, use of RAS blockers and use of other anti-hypertensive drugs. We did not adjust for age as age was already included in the built-in calculation of the SUDOSCAN-DKD score. We constructed receiver operating characteristic (ROC) curve to estimate the sensitivity and specificity of SUDOSCAN-DKD score for detecting CKD. The area under the ROC curve was calculated and the optimal cut-point was the peak of the curve where the sum of sensitivity and specificity was greatest. A 2-sided p-value <0.05 was considered significant. Statistical analysis was performed using Statistical Package for Social Science software (version 2.0, Chicago, Illinois, USA).

## Results

### Clinical Characteristics

Amongst these 2833 patients with type 2 diabetes (mean age: 58.6±6.4 years, 55.7% male, median duration of diabetes: 8 [inter-quartile range 3–14] years), 5.7% had CKD, 26.1% had microalbuminuria and 9.9% had macroalbuminuria. The mean estimated GFR was 42.1±14.0 ml/min/1.73m^2^ in the CKD group and 116.0±28.1 ml/min/1.73m^2^ in the non-CKD group (p = 0.001). Patients with CKD were older and had longer disease duration and worse metabolic profile with higher BMI, systolic BP, HbA_1c_ but lower blood haemoglobin (Table 1). They were also more likely to have microvascular and macrovascular complications and be treated with insulin, statins and RAS inhibitors.

**Table 1:** Clinical characteristics of 2833 Chinese patients with type 2 diabetes stratified by presence of chronic kidney disease.

	Patients with CKD (n = 163)	Patients without CKD (n = 2670)	p-value
Socio-demographics			
Age (years)	66.9 ± 9.2	58.1 ± 10.4	<0.001
Male (%)	54.6	55.7	0.764
Current/ex-smoker (%)	27.0	29.0	0.576
Metabolic parameters			
Disease duration (years)	15.8 ± 8.7	9.1 ± 7.2	<0.001
BMI (kg/m^2^)	27.1 ± 4.5	26.0 ± 4.3	0.004
Waist circumference (cm)			
Male	94.1 ± 9.8	92.5 ± 10.3	0.140
Female	93.7 ± 13.0	88.1 ± 11.4	<0.001
Systolic BP (mmHg)	145.8 ± 19.3	132.1 ± 17.1	<0.001
Diastolic BP (mmHg)	78.1 ± 9.7	78.3 ± 9.9	0.791
HbA_1c_ (%/mmol/mol)	7.8 ± 1.5 / 62 ± 11.9	7.4 ± 1.3 / 57 ± 10.0	<0.001
LDL-cholesterol (mmol/L)	2.2 ± 0.7	2.4 ± 0.8	0.003
HDL-cholesterol (mmol/L)	1.3 ± 0.4	1.3 ± 0.4	0.007
Triglyceride (mmol/L)	1.4 (1.1–2.0)	1.2 (0.9–1.8)	0.010
Haemoglobin (g/dL)	12.2 ± 1.9	13.7 ± 1.4	<0.001
Estimated GFR (ml/min/1.73m^2^)	42.1 ± 14.0	116.0 ± 28.1	0.004
Urine ACR (mg/mmol)	35.8 (7.6–180.8)	1.2 (0.5–4.1)	0.001
Diabetic complications			
Microalbuminuria (%)	30.2	23.2	0.042
Macroalbuminuria (%)	56.2	7.1	<0.001
Diabetic retinopathy (%)	35.6	19.3	<0.001
Sensory neuropathy (%)	11.7	2.9	<0.001
Coronary heart disease (%)	17.8	9.7	<0.001
Medication use			
ACE-inhibitors (%)	47.2	25.6	<0.001
ARB (%)	17.2	8.4	<0.001
Anti-hypertensive drugs (%)	71.8	40.8	<0.001
Statins (%)	58.3	38.2	<0.001
Insulin (%)	50.9	18.1	<0.001
SUDOSCAN			
Hand ESC	42.9 ± 22.1	54.6 ± 22.5	<0.001
Foot ESC	51.5 ± 21.7	62.0 ± 18.6	<0.001
SUDOSCAN-DKD Score	45.5 ± 12.3	58.6 ± 15.1	<0.001

mean±standard deviation, median (inter-quartile range), or percentages as appropriate

ACE, angiotensin-converting enzyme; ACR, albumin-to-creatinine ratio; ARB, angiotensin receptor blockers; BMI, body mass index; BP, blood pressure; CKD, chronic kidney disease; GFR, glomerular filtration rate; ESC, electrochemical skin conductance; HbA_1c_, glycated haemoglobin; HDL, high density-lipoprotein; LDL, low density-lipoprotein.

Electrochemical skin conductance was lower in the CKD group than the non-CKD group at both hands (42.9±22.1μS versus 54.6±22.5μS, p = 0.001) and feet (51.5±21.7μS versus 62.0±18.6μS, p = 0.001). The mean SUDOSCAN-DKD score were 45.5±12.3 and 58.6±15.1 in the CKD and non-CKD groups, respectively.

### Correlation of SUDOSCAN-DKD Score with Estimated GFR

**Fig 1:**
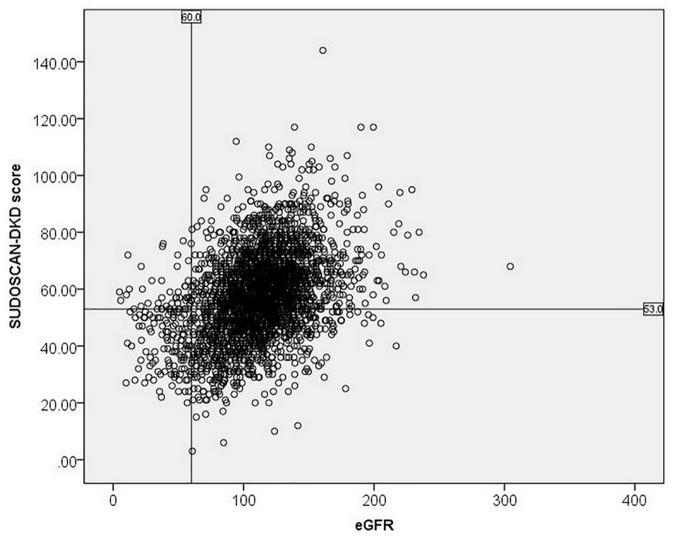
Scatterplot showing the relationship between SUDOSCAN-DKD score on y-axis and estimated glomerular filtration rate on x-axis.

The scatterplot shows the relationship between SUDOSCAN-DKD score and estimated GFR (Fig 1). On multiple linear regression, low SUDOSCAN-DKD score remained significantly associated with low estimated GFR (β-coefficient 0.334, p<0.001), in addition to long disease duration, male gender, high BMI, high systolic BP, low HbA_1c_, low LDL-cholesterol, and use of anti-hypertensive drugs (Table 2). Low SUDOSCAN-DKD score was also independently associated with high urine ACR although the magnitude of the correlation was small (β-coefficient -0.054, p = 0.005).

**Table 2:** Clinical factors associated with estimated glomerular filtration rate in Chinese patients with type 2 diabetes using multiple linear regression.

	Standardβ-coefficient	P value
Female gender	0.132	<0.001
Disease duration	-0.117	<0.001
BMI	-0.051	0.003
HbA_1c_	0.049	0.004
Systolic BP	-0.103	<0.001
LDL-cholesterol	0.018	0.288
Use of RAS blockers	-0.030	0.097
Use of anti-hypertensive drugs	-0.110	<0.001
SUDOSCAN-DKD score*	0.335	<0.001

BMI, body mass index; BP, blood pressure; HbA_1c_, glycated haemoglobin; LDL, low density-lipoprotein; RAS, renin-angiotensin system

*SUDOSCAN-DKD score included age and electrochemical skin conductance

### Performance of SUDOSCAN in Detecting CKD

**Fig 2:**
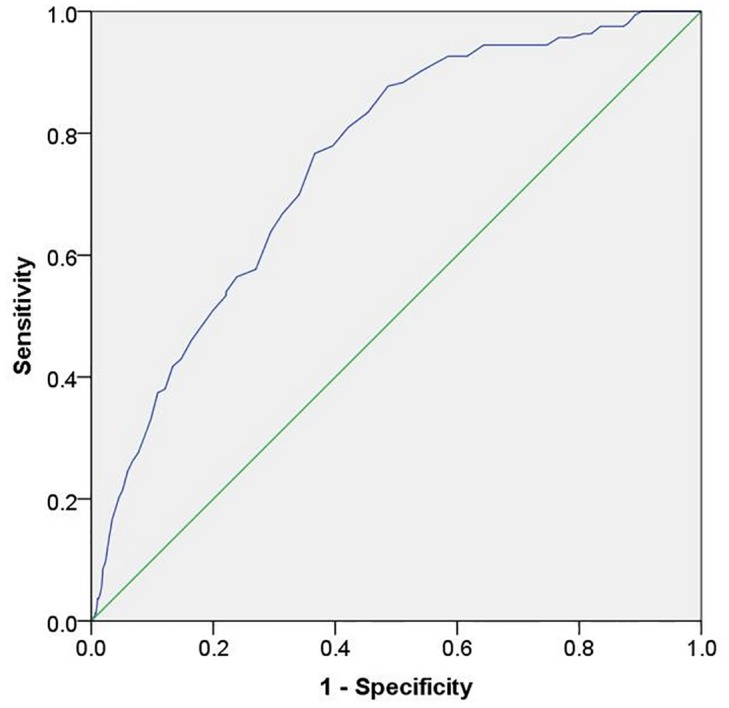
Receiver operating characteristic curve of SUDOSCAN-DKD score in detecting chronic kidney disease in Chinese patients with type 2 diabetes

The area under the ROC curve of SUDOSCAN-DKD score to predict CKD was 0.75 (95% confidence interval [CI] 0.72–0.79) of the total square (Fig 2). At SUDOSCAN-DKD score cut-off of 53, the test had 76.7 (95% CI 69.4–82.9)% sensitivity and 63.4 (95% CI 61.5–65.2)% specificity to detect CKD with positive predictive value (PPV) of 0.11 (95% CI 0.10–0.13) and negative predictive value (NPV) of 0.98 (95% CI 0.97–0.98). The likelihood ratio of a positive test was 2.09 (95% CI 1.89–2.30), while the negative likelihood ratio was 0.37 (95% CI 0.26–0.47). Using a higher SUDOSCAN-DKD score cut-off of 55 based on our group’s earlier analysis of 100 diabetic patients [17], the sensitivity was increased to 81.0 (95% CI 74.1–86.7)% while specificity was reduced to 57.9 (95% CI 56.0–59.8)%,

### Patients without CKD but Low SUDOSCAN-DKD Score

Among patients with no CKD (n = 2670), 36.7% were scored ≤53 on SUDOSCAN. Patients with low score were older and more likely to be female with longer duration of diabetes than those with score>53 (Table 3). These patients also had lower BMI, LDL-cholesterol, HbA_1c_, blood haemoglobin, estimated GFR but higher systolic BP and were more likely to have microalbuminuria, retinopathy, sensory neuropathy and cardiovascular disease than those with high score.

**Table 3:** Clinical characteristics of patients without chronic kidney disease stratified by SUDOSCAN-DKD score of 53.

	Patients with score ≤ 53 (n = 978)	Patients with score > 53 (n = 1692)	p-value
Socio-demographics			
Age (years)	67.2 ± 6.7	52.9 ± 8.3	<0.001
Male (%)	52.1	57.9	0.005
Current/ex-smoker (%)	30.9	28.0	0.111
Metabolic parameters			
Disease duration (years)	11.1 ± 8.0	8.0 ± 6.6	<0.001
BMI (kg/m^2^)	25.4 ± 4.0	26.4 ± 4.3	<0.001
Waist circumference (cm)			
Male	91.6 ± 10.0	93.0 ± 10.4	0.011
Female	87.6 ± 10.6	88.4 ± 11.8	0.248
Systolic blood pressure (mmHg)	136.3 ± 17.6	129.6 ± 16.3	<0.001
Diastolic blood pressure (mmHg)	77.1 ± 9.8	78.9 ± 9.9	<0.001
HbA_1c_ (%/mmol/mol)	7.3 ± 1.2 / 56 ± 9.2	7.5 ± 1.5 / 58 ± 11.6	0.001
LDL-cholesterol (mmol/L)	2.2 ± 0.7	2.5 ± 0.8	<0.001
HDL-cholesterol (mmol/L)	1.4 ± 0.4	1.3 ± 0.4	0.055
Triglyceride (mmol/L)	1.2 (0.9–1.6)	1.3 (0.9–1.8)	0.003
Haemoglobin (g/dL)	13.4 ± 1.4	13.9 ± 1.4	<0.001
Estimated GFR (ml/min/1.73m^2^)	105.4 ± 25.9	122.1 ± 27.6	<0.001
Urine ACR (mg/mmol)	1.5 (0.6–5.5)	1.1 (0.5–3.2)	0.001
Diabetic complications			
Microalbuminuria (%)	28.8	24.0	<0.001
Macroalbuminuria (%)	8.4	6.4	0.059
Diabetic retinopathy (%)	23.4	20.3	<0.001
Sensory neuropathy (%)	4.5	2.0	<0.001
Coronary heart disease (%)	14.0	7.2	<0.001
Medication use			
ACE-inhibitors (%)	26.7	25.0	0.336
ARB (%)	10.8	6.9	<0.001
Anti-hypertensive drugs (%)	53.5	37.5	<0.001
Statins (%)	41.5	36.2	0.007
Insulin (%)	17.8	18.3	0.761
SUDOSCAN			
Hand ESC	43.4±21.3	61.0±20.6	<0.001
Foot ESC	49.6±18.5	69.1±14.5	<0.001
SUDOSCAN Score	43.5 ± 8.1	67.2 ± 10.8	<0.001

mean±standard deviation, median (inter-quartile range), or percentages as appropriate

ACE, angiotensin-converting enzyme; ACR, albumin-to-creatinine ratio; ARB, angiotensin receptor blockers; BMI, body mass index; GFR, glomerular filtration rate; ESC, electrochemical skin conductance; HbA_1c_, glycated haemoglobin; HDL, high density-lipoprotein; LDL, low density-lipoprotein.

## Discussion

Diabetic kidney disease is prevalent and requires periodic laboratory assessments to allow timely intervention for cardiovascular-renal protection. In this large cross-sectional sample of community-dwelling Chinese patients with type 2 diabetes, patients with a low SUDOSCAN-DKD score (≤53) had 2-fold increased likelihood of having CKD than those with higher scores. Based on our findings, we conclude that SUDOSCAN may be useful in detecting patients at risk of having CKD. The worse risk factor and complications profiles in patients with normal GFR but low SUDOSCAN-score suggested that the device might also have a utility in detecting early renal involvement although prospective evaluation will be needed. Given the non-invasive and portable nature of SUDOSCAN, it can be a used in outreach programs or low resource setting as part of a CKD screening program.

Sudomotor function is a subtype of autonomic function which reflects the integrity of sympathetic nerve fibers innervating the sweat glands [10]. Sudomotor dysfunction in diabetes is manifested by attenuated sweat response to high ambient temperature and humidity especially in the lower peripheries [10]. With longstanding diabetes, chronic impairment of sudomotor function translates to reduced suppleness of skin, dryness and fissures especially in their feet. The C fibers which innervate the sweat glands are typically long, thin and poorly myelinated making them highly susceptible to damages by metabolic processes. The density of nerve fibres innervating sweat gland based on skin biopsies has been shown to correlate with measures of glycaemia including HbA_1c_ in patients with diabetes [21]. Moreover, damages to these nerves may have occurred even before the onset of overt diabetes as supported by reports of sudomotor dysfunction in subjects with impaired glucose tolerance and metabolic syndrome [11–13,22].

Sudomotor function *per se*, however, is rarely measured in routine clinical practice as its precise assessment is both time- and labor-consuming [23]. SUDOSCAN offers a rapid, easy, non-invasive and validated tool to evaluate sudomotor function in a busy clinic setting. Given the causal role of dysglycemia in impaired sudomotor function [11–13], other researchers have used the same device with different built in algorithms (EZSCAN) to detect pre-diabetes in European, Indian and Chinese populations [12–15].

Graded relationships between low electrochemical skin conductance measured by SUDOSCAN and severity of peripheral sensory neuropathy have been reported in people with diabetes [24,25]. The use of SUDOSCAN to predict microvascular complications other than neuropathy has also been explored in several small studies. From our group, analysis of 50 patients with and 50 patients without DKD showed that electrochemical skin conductance was associated with GFR independent of age, gender, disease duration, smoking, alcohol, BP, triglyceride, hemoglobin, presence of other microvascular complications, and use of medications including RAS inhibitors and anti-hypertensive agents [17]. Freedman and colleagues studied 390 African and European Americans with type 2 diabetes and 166 controls and found independent association between skin conductance and GFR (but not urine ACR) in African but not European Americans [26]. In the present cohort, we were able to confirm the independent association between SUDOSCAN-DKD score and estimated GFR. Sudomotor dysfunction may have similar pathogenic mechanisms to DKD. Processes downstream to sustained hyperglycaemia including activation of protein kinase C, activation of the polyol pathway, and formation of advanced glycosylation end products that are known to drive diabetic renal changes, have also been implicated in causing reduction of endoneurial blood flow and direct nerve injury [27].

The natural progression of DKD involves the gradual transition from hyperfiltration to albuminuria, to decline in GFR. While microalbuminuria is traditionally viewed as an early indicator of diabetic renal involvement, its predictive value for advance renal disease is recently challenged by poor sensitivity and specificity [28]. On the one hand, normoalbuminuria does not preclude the presence of renal parenchymal changes and between 20–70% of patients may progress to significant renal impairment while maintaining normoalbuminuria [28,29]. On the other hand, albumin excretion may be increased in response to pathological or physiological processes unrelated to diabetes such as posture, exercise, puberty, smoking, obesity, and infection. In addition, spontaneous remission of microalbuminuria has been observed in 20–60% of patients without specific anti-proteinuric therapy [28]. The statistically significant but numerically small association between SUDOSCAN-DKD score and urine ACR in the present study might be due to its single measurement with marked inter- and intra-individual variations. The use of RAS inhibitors which have been shown to effectively lower urine ACR might also have confounded the relationship between SUDOSCAN-DKD score with urine ACR.

In this analysis, the optimal SUDOSCAN-DKD score for detecting CKD was 53 with 77% sensitivity and 63% specificity. In our previous case-control cohort, the cutoff value of 55 had 94% sensitivity and 78% specificity. The better performance might be due to greater case dichotomization in our previous study [17]. While these subtle differences in performance might be due to study design, selection criteria, sample size and ethnicity, the overall evidence supports the clinical utility of SUDOSCAN in discriminating subjects with CKD.

We acknowledge the following limitations. One, despite the relatively large sample size of this cross-sectional cohort, prospective evaluation is needed to confirm the clinical utility of SUDOSCAN for risk stratification and prognostication in CKD. Two, only Chinese patients were studied and our results might not be generalizable to other ethnic groups which might have different patterns of risk attributes. Three, SUDOSCAN is a screening instrument only and cannot replace blood and urine sampling for quantification of renal function.

## Conclusion

In conclusion, SUDOSCAN may detect patients at high risk of CKD. Given its non-invasive, portable and easy-to-use features, it may be used in outreach programs or low resource settings as part of a detection program to identify high risk subjects for further assessments.
